# Determinants of non-vaccination against seasonal influenza in Canadian adults: findings from the 2015–2016 Influenza Immunization Coverage Survey

**DOI:** 10.17269/s41997-018-0018-9

**Published:** 2018-03-30

**Authors:** Noushon Farmanara, Lindsey Sherrard, Ève Dubé, Nicolas L. Gilbert

**Affiliations:** 1 Centre for Immunization and Respiratory Infectious Diseases, Public Health Agency of Canada, Ottawa, ON Canada; 2 Department of Epidemiology, Biostatistics and Occupational Health, McGill University, Montréal, QC Canada; 3 Institut National de Santé Publique du Québec, Québec, QC Canada

**Keywords:** Influenza vaccine, Surveys and questionnaires, Immunization programs, Canada, Adults, Vaccine antigrippal, Enquêtes et questionnaires, Programmes de vaccination, Canada, Adultes

## Abstract

**Objectives:**

The study objectives were to (1) identify determinants of non-vaccination against seasonal influenza in Canadian adults and (2) examine self-reported reasons for non-vaccination.

**Methods:**

The data source was the 2015–2016 Influenza Immunization Coverage Survey, a national telephone survey of Canadian adults. Participants (*n* = 1950) were divided into three groups: adults aged 18–64 years with (*n* = 408) and without (*n* = 1028) chronic medical conditions (CMC) and adults ≥ 65 years (*n* = 514). Logistic regression was used to measure associations between sociodemographic factors and non-vaccination for the 2015–2016 influenza season. Weighted proportions were calculated to determine the main self-reported reasons for not receiving the influenza vaccine.

**Results:**

Younger age was found to be associated with non-vaccination across all groups. In adults ≥ 65 years, elementary- or secondary- vs. university-level education (aOR 1.87, 95% CI 1.14-3.06) was also significantly associated with non-vaccination. Significant variation in vaccine uptake was found for several sociodemographic factors in adults aged 18–64 without CMC. Low perceived susceptibility or severity of influenza and lack of belief in the vaccine’s effectiveness were the most commonly reported reasons for not receiving the vaccine.

**Conclusion:**

In general, our results were consistent with findings from other Canadian and American studies on seasonal influenza vaccine uptake. Belief that the influenza vaccine is not needed was common, even among those at increased risk of influenza-related complications. Additional research is needed to better understand how sociodemographic factors such as income and education may influence uptake and to raise awareness of potential complications from influenza infection in high-risk adults.

## Résumé

**Objectifs:**

Les objectifs de cette étude étaient : 1) identifier les déterminants de la non-vaccination contre la grippe chez les adultes canadiens; 2) examiner les raisons auto-déclarées de ne pas s’être fait vacciner.

**Méthodes:**

Les données provenaient de l’Enquête sur la couverture vaccinale antigrippale de 2015–2016, un sondage téléphonique réalisé chez des adultes canadiens. Les 1950 participants ont été divisés en trois groupes : les 18 à 64 ans avec (*n* = 408) ou sans problème de santé chronique (PSC) (*n* = 1028) et les 65 ans et plus (*n* = 514). Des régressions logistiques ont été utilisées pour mesurer les associations entre les facteurs sociodémographiques et la non-vaccination pour la saison grippale de 2015–2016. Des proportions pondérées ont été calculées pour identifier les principales raisons auto-déclarées de ne pas s’être fait vacciner.

**Résultats:**

Le jeune âge était associé à la non-vaccination dans les trois groupes étudiés. Chez les 65 ans et plus, n’avoir complété que des études primaires ou secondaires, comparativement à des études universitaires, était aussi associé à la non-vaccination (RCa 1,87, IC 95%: 1,14 à 3,06). Des associations significatives avec les niveaux de vaccination ont été trouvées pour plusieurs facteurs sociodémographiques chez les 18–64 ans sans PSC. Les perceptions d’un faible risque d’attraper la grippe et d’une faible gravité de celle-ci, ainsi que le manqué de confiance en l’efficacité du vaccine étaient les raisons les plus fréquemment rapportées de ne pas s’être fait vacciner.

**Conclusion:**

En général, nos résultats sont similaires à ceux d’autres études canadiennes ou américaines sur la vaccination contre la grippe saisonnière. La perception que le vaccin antigrippal n’est pas nécessaire était courante, y compris chez les populations à risque accru de complication. Des recherches plus poussées seraient nécessaires pour comprendre l’association entre d’une part des facteurs sociodémographiques comme le revenu et l’éducation, et d’autre part la vaccination antigrippale, et pour sensibiliser les adultes à risque aux complications possibles de la grippe.

## Introduction

In Canada, it is estimated that 10–20% of the population become infected with influenza every year (Public Health Agency of Canada 2015). Furthermore, it is estimated that an average of 12,200 hospitalizations and 3500 deaths per year are attributable to influenza (Schanzer et al. 2008, 2013). The National Advisory Committee on Immunization (NACI) recommends that all individuals 6 months of age or older and without contraindications be vaccinated against influenza (Public Health Agency of Canada 2015). Those considered by NACI to be at high risk of influenza-related complications include those with certain medical conditions and those aged 65 years or older (Public Health Agency of Canada 2015).

In 2013–2014, rates of vaccination against influenza were estimated to be 64% among adults aged 65 years and over and 32% in those aged 18–64 years with one or more chronic conditions (Gionet 2015). These rates fall short of influenza vaccination coverage goals of 80% for adults aged 65 years or more and for adults aged 18–64 years with high-risk conditions set at the 2005 National Consensus Conference for Vaccine-Preventable Diseases in Canada (Public Health Agency of Canada 2007). Accordingly, the rates of non-vaccination in these high-risk groups were 36% among adults 65 years and over, and 68% in those aged 18–64 years with chronic conditions during this period. Compared to previous years, these rates remain stable, despite the fact that all provincial and territorial governments fund the influenza vaccine for individuals at greater risk of influenza infection and related complications (Government of Canada 2016). Previous studies have aimed to identify possible determinants of vaccination and non-vaccination against influenza among various adult groups (Takayama et al. 2012; Endrich et al. 2009; Linn et al. 2010; Andrew et al. 2004; Chen et al. 2007). However, apart from age and chronic disease status, studies on the associations between sociodemographic factors and influenza vaccination have had conflicting results. A systematic review of factors associated with influenza uptake in adults aged 18–64 years found that the association between education level or income and uptake of the influenza vaccine was inconsistent across the 21 studies from Asia, Europe, the United States, and Australia (Yeung et al. 2016). Knowledge, attitudes, and beliefs (KAB) surrounding influenza vaccination have also been documented. One meta-analysis based on 14 years of influenza-related communications from the US CDC found that a lack of perceived susceptibility and severity and lack of belief in the effectiveness of the influenza vaccine were the most common themes arising from in-depth interviews, focus groups, and surveys (Nowak et al. 2015). However, fewer studies have looked at influenza-related KABs in the Canadian context, in particular, nationally (Dubé et al. 2014; Guthrie et al. 2017; Kwong et al. 2007). This study provides recent national data for Canada, based on the results of the Influenza Immunization Coverage Survey for the 2015–2016 influenza season. Measuring influenza vaccine uptake and behaviours yearly is important for measuring progress towards Canada’s national vaccination coverage goals and to inform decision-making and future influenza vaccination  programs (Public Health Agency of Canada 2017). To the best of our knowledge, results from this survey provide a national perspective that is better aligned to the Canadian context, providing important information on chronically ill groups that are aligned with NACI-identified risk groups and recommendations (Public Health Agency of Canada 2015).

The objectives of this study were to:

1. Identify sociodemographic determinants of non-vaccination against influenza in adults; and
2. Examine self-reported reasons for non-vaccination against seasonal influenza.

## Methods

### Survey administration and content

The 2015–2016 Influenza Immunization Coverage Survey was conducted by Léger Marketing on behalf of the Public Health Agency of Canada (PHAC). The survey was a national telephone survey of 2000 Canadian adults conducted between January 25th and February 22nd, 2016. Phone numbers were randomly selected from lists of Canadian numbers, with quotas for provinces or territories, and for dwelling type (landline or cell-only) within regions. Interviews were conducted using computer-assisted telephone interviewing (CATI). Up to eight call-back calls were allowed for each number over the fieldwork window and fixed call-back appointments were made when possible. Callbacks to a given number were made at various times of the day or evening and various days of the week, including weekends. One adult respondent per household was surveyed to ascertain vaccination status, demographic information, and other details related to influenza vaccination in the 2015–2016 season. Adult respondents were asked whether they had received the seasonal influenza vaccine since September 1st, 2015 and were coded as non-vaccinated if they responded “no” and vaccinated if they responded “yes.” Using an open-ended format, non-vaccinated respondents were asked to provide reasons for not receiving the vaccine.

### Variables

The dependent variable was vaccination status, where non-vaccination was defined as not having received the influenza vaccine during the 2015–2016 influenza season. Respondents who refused to respond or did not know whether they had received the vaccine were excluded from the dataset. Independent variables considered in the analysis were age, sex, presence or absence of a chronic medical condition (CMC) associated with higher risk of complication from influenza (Public Health Agency of Canada 2015), education level, income, country of birth, province or territory of residence, and the first language learned. To ascertain presence or absence of a CMC, participants were asked if they had been diagnosed with any of the conditions associated with a higher risk of complications from influenza infection, as defined by NACI (Public Health Agency of Canada 2015). Survey respondents who reported having any one or more of these conditions were considered to have a CMC.

Provinces and territories of residence were combined into categories based on public funding of the influenza vaccine. The influenza vaccine is provided free of charge in all provinces and territories for adults with high-risk CMC and those aged 65 years or older. However, three provinces (British Columbia (BC), New Brunswick (NB), and Québec (QC)) do not provide the influenza vaccine free of charge for adults aged 18–64 years without CMC (Public Health Agency of Canada 2015; Government of Canada 2016). In addition, in QC, the vaccine is provided free of charge to all adults aged 60 years or older as opposed to 65 years or older. In order to explore possible associations between vaccine uptake and differences in public funding of the influenza vaccine for adults, three categories were created for the analysis (1—BC and NB, 2—QC, and 3—all other provinces and territories).

### Analytic methods

The analysis of adults was categorized into three groups: adults aged 18–64 years with and without CMC and adults aged 65 years or older. Respondents whose age was unknown (*n* = 38) and those aged 18–64 years whose CMC status was unknown (*n* = 12) were excluded from the analysis. For each group, weighted proportions were calculated to determine rates of non-vaccination and the main self-reported reasons for not receiving the influenza vaccine. Logistic regression was used to estimate unadjusted odds ratios (OR) and adjusted OR (aOR) and their 95% confidence intervals. Factors with a *p* value of < 0.1 in simple logistic regressions were included in multiple regressions. Results of the survey were weighted by age, gender, region, language, and presence of minor children in the household. Cell-only users were estimated to represent approximately 21% of the Canadian population (Statistics Canada 2013). Therefore, the weight of respondents who were categorized as cell-only users was adjusted to correspond to this 21% proportion after weighting. Sampling weights were derived from the Statistics Canada 2011 national census. Statistical analyses were conducted using SAS (Enterprise Guide 5.1.). Immunization coverage surveys are part of PHAC’s routine health surveillance activities and, as such, do not require ethics approval. Participation in the survey was entirely voluntary and no identifying information (e.g., name, date of birth, address) was collected.

## Results

The overall response rate for the survey was 19%. The sociodemographic characteristics of the survey population (*n* = 1950) are shown in Table 1. Overall, rates of non-vaccination were 75.6% (95% CI 72.6–78.6) for adults 18–64 years without CMC (*n* = 1028) (Table 2). For adults 18–64 years with CMC (*n* = 408), for whom the influenza vaccine is recommended, the rate of non-vaccination was 62.8% (95% CI 57.5–68.1) (Table 3). Finally, the rate of non-vaccination against influenza was 35.4% (95% CI 31.1–39.8) for adults aged 65 years or more (*n* = 514) (Table 4). For adults aged 65 years or more with CMC, rates of non-vaccination were lowest at 27.4% (95% CI 22.1–32.8).

**Table 1:** Characteristics of the study sample of Canadian adults (*n* = 1950), 2015–2016

Variable	*n*	Study population (%)
Age		
18–44	658	33.7
45–64	778	39.9
65–70	209	10.7
> 70	305	15.6
Chronic medical condition		
No	1240	63.9
Yes	702	36.1
Missing/refusal	8	
Gender		
Male	797	40.9
Female	1153	59.1
Education		
Elementary or secondary	682	35.6
College graduate	564	29.4
University graduate	670	35.0
Missing/refusal	34	
Income		
Less than $40,000	442	28.4
$40,000 to $59,999	290	18.6
$60,000 to $99,999	394	25.3
$100,000 or more	431	27.7
Missing/refusal	393	
Country of birth		
Canada	1596	82.1
Other (> 15 years in Canada)	225	11.6
Other (≤ 15 years in Canada)	124	6.4
Missing/refusal	5	
P/T* by public funding of influenza vaccine^†^		
BC and NB (not universal)	314	16.1
QC (not universal)	488	25.0
Rest of Canada (universal program)	1148	58.9
First language^‡^		
French	491	25.2
English	1196	61.4
Other	261	13.4
Missing/refusal	2	

*Province/territory

^†^Influenza vaccine is not free for those 18–64 years without CMC in BC/NB, and 18–59 years without CMC in QC

^‡^Respondents who learned both French and English as a first language were included in the English category

**Table 2:** Rates and determinants of non-vaccination for seasonal influenza in Canadian adults 18–64 without CMC

Variable	Valid respondents	Rate (95% CI)	Unadjusted OR (95% CI)	Adjusted OR^§^ (95% CI)
Overall	1028	75.6 (72.6–78.6)		
Age				
18–44	517	78.5 (74.4–82.6)	1.45 (1.06–2.00)	1.48 (1.05–2.07)
45–64	511	71.5 (67.2–75.7)	Reference	Reference
Gender				
Male	453	80.0 (75.9–84.1)	1.64 (1.18–2.28)	1.47 (1.05–2.06)
Female	575	71.0 (66.6–75.4)	Reference	Reference
Education				
Elementary or secondary	285	76.8 (70.9–82.7)	1.31 (0.88–1.97)	1.43 (0.91–2.25)
College graduate	321	80.6 (75.7–85.5)	1.64 (1.11–2.43)	1.75 (1.16–2.63)
University graduate	410	71.6 (66.8–76.4)	Reference	Reference
Income				
$0 to $39,999	153	79.4 (71.9–87.0)	1.51 (0.89–2.58)	1.01 (0.55–1.84)
$40,000 to $59,999	149	86.4 (80.6–92.2)	2.50 (1.42–4.39)	2.14 (1.19–3.85)
$60,000 to $79,999	128	73.0 (64.1–81.8)	1.06 (0.63–1.78)	0.85 (0.49–1.46)
$80,000 to $99,999	112	79.8 (71.7–87.8)	1.55 (0.88–2.72)	1.41 (0.79–2.51)
$100,000 or more	313	71.8 (66.3–77.4)	Reference	Reference
Country of birth				
Canada	835	73.9 (70.5–77.3)	Reference	Reference
Other country	193	80.7 (74.4–86.9)	1.47 (0.96–2.27)	1.61 (1.01–2.56)
P/T^*^ by public funding of influenza vaccine^†^				
BC and NB (not universal)	168	71.8 (63.8–79.8)	0.91 (0.59–1.42)	0.91 (0.57–1.46)
QC (not universal)	256	83.8 (79.0–88.7)	1.86 (1.23–2.81)	1.82 (1.19–2.80)
Rest of Canada (universal program)	604	73.6 (69.6–77.6)	Reference	Reference
First language^‡^				
French	246	83.8 (78.9–88.7)	1.99 (1.32–2.99)	
English	631	72.2 (68.3–76.1)	Reference	
Other	151	77.1 (69.4–84.8)	1.30 (0.81–2.08)	

Rates and odds ratios are weighted

*CMC* chronic medical condition

^*^Province/territory

^†^Influenza vaccine is not free for those 18–64 years without CMC in BC/NB, and 18–59 years without CMC in QC

^‡^Respondents who reported both French and English were included in the English category

^§^Adjusted for age, gender, education level, income, country of birth, and province/territory by coverage

**Table 3:** Rates and determinants of non-vaccination for seasonal influenza in Canadian adults 18–64 with CMC

Variable	Valid respondents	Rate (95% CI)	Unadjusted OR (95% CI)
Overall	408	62.8 (57.5–68.1)	
Age			
18–44	141	76.4 (68.2–84.5)	2.85 (1.70–4.79)
45–64	267	53.1 (46.5–59.7)	Reference
Gender			
Male	149	67.0 (58.7–75.4)	1.37 (0.85–2.19)
Female	259	59.8 (52.9–66.7)	Reference
Education			
Elementary or secondary	161	69.1 (61.2–77.1)	1.41 (0.80–2.51)
College graduate	137	56.3 (46.8–65.9)	0.81 (0.45–1.46)
University graduate	107	61.3 (50.8–71.8)	Reference
Income			
$0 to $39,999	103	68.9 (58.7–79.1)	1.60 (0.87–2.97)
$40,000 to $79,999	112	61.4 (51.1–71.7)	1.15 (0.64–2.06)
$80,000 or more	128	58.0 (48.3–67.7)	Reference
Country of birth			
Canada	351	64.3 (58.6–70.0)	Reference
Other country	57	55.0 (40.4–69.6)	0.68 (0.36–1.27)
P/T^*^ by public funding of influenza vaccine^†^			
BC and NB (not universal)	50	54.2 (37.5–70.9)	0.76 (0.38–1.55)
QC (not universal)	103	71.7 (62.2–81.3)	1.64 (0.95–2.82)
Rest of Canada (universal program)	255	60.8 (54.0–67.6)	Reference
First language^‡^			
French	105	70.5 (61.2–79.7)	1.55 (0.92–2.61)
English	256	60.6 (53.9–67.4)	Reference
Other	47	60.4 (44.0–76.8)	0.99 (0.48–2.04)

Rates and odds ratios are weighted

*CMC* chronic medical condition

^*^Province/territory

^†^Influenza vaccine is not free for those 18–64 years without CMC in BC/NB, and 18–59 years without CMC in QC

^‡^Respondents who reported both French and English were included in the English category

**Table 4:** Rates and determinants of non-vaccination for seasonal influenza in adults ≥ 65 years

Variable	Valid respondents	Rate (95% CI)	Unadjusted OR (95% CI)	Adjusted OR^§^ (95% CI)
Overall	514	35.4 (31.1–39.8)		
Age				
65–70	209	44.7 (37.6–51.9)	2.00 (1.36–2.94)	1.79 (1.19–2.69)
> 70	305	28.8 (23.5–34.1)	Reference	Reference
Gender				
Male	195	38.3 (31.2–45.4)	1.26 (0.85–1.84)	
Female	319	33.1 (27.7–38.5)	Reference	
Education				
Elementary or secondary	236	38.2 (31.6–44.7)	1.58 (0.99–2.51)	1.87 (1.14–3.06)
College graduate	106	40.6 (30.6–50.6)	1.75 (1.01–3.04)	1.71 (0.97–3.03)
University graduate	153	28.1 (20.5–35.7)	Reference	Reference
Income				
$0–$39,999	186	36.1 (28.9–43.4)	1.10 (0.70–1.75)	
$40,000 or more	173	33.9 (26.3–41.5)	Reference	
Country of birth				
Canada	403	34.0 (29.2–38.9)	Reference	
Other country	104	37.8 (27.7–48.0)	1.18 (0.73–1.90)	
Chronic medical condition				
No	212	46.1 (38.9–53.2)	2.26 (1.53–3.34)	2.19 (1.46–3.29)
Yes	294	27.4 (22.1–32.8)	Reference	Reference
P/T^*^ by public funding of influenza vaccine^†^				
BC and NB (not universal)	96	40.5 (29.9–51.1)	1.46 (0.88–2.42)	
QC (not universal)	129	40.1 (31.3–48.8)	1.43 (0.91–2.24)	
Rest of Canada (universal program)	289	31.9 (26.1–37.6)	Reference	
First language^‡^				
French	140	41.0 (32.7–49.4)	1.59 (1.03–2.43)	1.57 (0.99–2.46)
English	309	30.5 (25.1–35.9)	Reference	Reference
Other	63	43.2 (30.1–56.3)	1.73 (0.97–3.10)	1.69 (0.93–3.06)

Rates and odds ratios are weighted

^†*^Province/territory

^†^Influenza vaccine is not free for those 18–64 years without CMC in BC/NB, and 18–59 years without CMC in QC

^‡^Respondents who reported both French and English were included in the English category

^§^Adjusted for age, education level, CMC status, and first language learned

### Determinants of non-vaccination

Among adults aged 18–64 years without CMC, being male, younger age (18–44 vs. 45–64 years), having a college-level education (as compared to university-level), having a reported household income of $40,000 to $59,999 (as compared to $100,000 or more), being born outside of Canada, and residing in QC (as compared to the rest of Canada, excluding BC and NB) were found to be independent predictors of non-vaccination against influenza (Table 2). There was no statistically significant difference found between elementary- or secondary-level education as compared to university level in the odds of non-vaccination. Despite statistical significance in the simple regression, the first language was not included in the multiple logistic regression model because of its strong association with the variable for province or territory of residence by public funding of the influenza vaccine.

In adults aged 18–64 years with CMC, younger age (18–44 vs. 45–64 years) was again found to be an independent predictor of non-vaccination against influenza (Table 3). No other factors analyzed were significant in the simple logistic regressions; therefore, no multiple regression was carried out.

In adults aged 65 years or more, again, younger age and the absence of CMC were significant predictors of non-vaccination against influenza. Non-vaccination was also significantly higher in participants with elementary- or secondary-level education as compared to university graduates (Table 4).

### Self-reported reasons for non-vaccination

The most commonly reported reasons for non-vaccination were not needing it/not high risk/not recommended, lack of time, and lack of belief in the vaccine’s effectiveness (Table 5). The same reasons were consistently reported across all adult groups, including those at greater risk of influenza-related complications.

**Table 5:** Main reasons for not receiving seasonal influenza vaccine during the 2015–2016 vaccination campaign for Canadian adults

	Age group (years)		
	18–64 without CMC^*^	18–64 with CMC^†^	≥ 65^‡^
Number of valid respondents	728	239	169
Reasons for not receiving influenza vaccine	% (95% CI)	% (95% CI)	% (95% CI)
Allergic to vaccine	0.8 (0.1–1.4)	6.4 (3.0–9.8)	5.3 (1.8–8.8)
Dislike/fear of needles	1.6 (0.3–2.9)	2.1 (0.2–4.1)	2.1 (0.0–4.3)
Don’t need it/not high risk/not recommended	*46.0* (*41.8–50.2*)	*35.8* (*28.7–42.8*)	*41.2* (*33.3–49.2*)
Lack of information	2.1 (0.7–3.5)	0.1 (0.0–0.3)	2.0 (0.0–4.3)
No time	*17.6* (*14.1–21.1*)	*17.1* (*11.5–22.7*)	*11.2* (*6.0–16.4*)
Difficulty getting to a clinic/doc’s office	0.6 (0.0–1.2)	1.1 (0.0–2.1)	3.3 (0.0–7.0)
Do not believe in the vaccine’s effectiveness	*21.7* (*18.2–25.2*)	*26.4* (*20.0–32.8*)	*27.5* (*20.6–34.5*)
Believe vaccines are unsafe (risks associated)	4.0 (2.3–5.7)	4.3 (1.2–7.4)	5.4 (1.8–9.0)
Fear side effects of vaccine	4.0 (2.4–5.6)	7.1 (3.6–10.6)	5.3 (1.8–8.9)
Was sick/in the hospital	1.1 (0.4–1.8)	1.4 (0.0–2.9)	4.4 (1.5–7.3)
Non-specific/chose not to	2.1 (0.9–3.4)	2.0 (0.1–3.8)	0.8 (0.0–2.0)
Did not want it	2.3 (1.1–3.4)	1.2 (0.0–2.5)	0.9 (0.0–2.5)
Never received the vaccine	1.6 (0.5–2.6)	2.0 (0.0–4.5)	0.6 (0.0–1.8)
Other	0.9 (0.3–1.6)	0.9 (0.0–1.9)	3.1 (0.3–5.8)

Reasons that represented less than 2% of valid responses across all groups are not shown above

^*^32 respondents (18–64 years without CMC) did not give a reason for not receiving the influenza vaccine

^†^12 respondents (18–64 years with CMC) did not give a reason for not receiving the influenza vaccine

^‡^12 respondents (≥ 65 years) did not give a reason for not receiving the influenza vaccine

## Discussion

In 2015–2016, rates of non-vaccination far exceeded the maximum of 20% that would be needed to achieve national goals of 80% influenza vaccine coverage for high-risk groups such as adults aged 18–64 years with CMC and adults aged 65 years or older. Rates of non-vaccination among Canadian adults aged 18–64 years with (62.8%) and without (75.6%) CMC were somewhat similar to estimates from the US during the 2014–2015 influenza season (52.4 and 64.5%, respectively) (Centers for Disease Control and Prevention (CDC) 2016). Rates of non-vaccination in Canadian adults aged 65 years or older during the 2015–2016 influenza season (35.4%) were similar to those from the US in 2014–2015 (34.1%) (Centers for Disease Control and Prevention (CDC) 2016).

### Determinants of non-vaccination against influenza

This analysis identified significant sociodemographic variations in the odds of non-vaccination in Canadian adults. The finding that adults in younger age categories had a higher odds of non-vaccination was maintained across all groups, including among adults with CMC that place them at greater risk of influenza-related complications. This is consistent with other studies that have found the rate of influenza vaccination to increase with age (Takayama et al. 2012; Endrich et al. 2009; Kwong et al. 2007; Polisena et al. 2012). The perception that the influenza vaccine is not needed is an important deterrent against the uptake of the influenza vaccine, even among the high-risk adults. Therefore, there may be a need for health professionals offering or promoting the vaccine to address beliefs that the vaccine is not needed in this high-risk group, and to dispel the perception that the influenza vaccine is for seniors only.

Results of this survey are consistent with findings from a Canadian study (Kwong et al. 2007) and an American study (Takayama et al. 2012) that found sex differences in the uptake of the influenza vaccine. Kwong et al. found lower uptake of influenza vaccination among males aged 12 years and older (Kwong et al. 2007). Two studies in the US using data from the 2008 and 2009 cycles of the Behavioral Risk Factor Surveillance System found significantly lower uptake of the influenza vaccine in males aged 18–64 years (Takayama et al. 2012) and 50–64 years (Linn et al. 2010), and no sex-based differences in adults 65 years and older. This is consistent with our study results that found no sex-based differences in influenza vaccine uptake in adults 65 years or older, and with other studies from Canada and the US (Takayama et al. 2012; Andrew et al. 2004). In contrast, another study of 11 European countries found either a higher uptake of influenza vaccination in males as compared to females or no significant sex differences (Endrich et al. 2009). Lower influenza vaccine uptake in young males may be related to lower health service utilization in comparison to females and may (in part) explain why differences are not frequently observed among older adults who have greater contact with the health care system (Linn et al. 2010).

In our analysis, the only subgroup for which we found a difference between foreign-born and Canadian-born participants was in adults aged 18–64 years without CMC. Other studies in Canada and Europe have found evidence of lower influenza vaccine uptake among immigrants; although, differences in study population may limit the generalizability of these findings (Hobbs and Buxton 2014; Jiménez-García et al. 2008a, b). These studies have suggested that lower influenza vaccine coverage among immigrants may be related to other non-financial barriers (e.g., cultural, language) that can negatively impact access to preventive services such as vaccination (Jiménez-García et al. 2008a, b). However, lack of statistical power in the current study sample did not allow us to account for the length of time foreign-born participants had spent in Canada nor the impact of other culturally related barriers to vaccination. Therefore, although we observed statistically significant differences in adults 18–64 years without CMC, additional research would be needed to determine whether these differences are consistently observed and to identify the underlying reasons for this variation.

Varying associations between education or income and non-vaccination were seen across the adult groups in our analyses, both in significance and nature of the association. One German study found that education level was not significantly associated with influenza vaccine uptake in adults aged 18–59 years with CMC, which is consistent with the results of this survey and another American study (Takayama et al. 2012). The same study found that higher education was associated with lower vaccine uptake among adults aged 60 years or more, whereas in our survey, rates of non-vaccination were higher among older adults with lower education. Higher uptake in older adults with higher education levels was also found in a large Canadian survey, the Canadian Study of Health and Aging (Andrew et al. 2004). Although in adults aged 18–64 years without CMC, college graduates had higher rates of non-vaccination than university graduates in our analysis, there was no clear relationship between increasing or decreasing levels of education and vaccination status.

Similarly, associations between income and influenza vaccine uptake have also been inconsistent in the literature (Takayama et al. 2012; Endrich et al. 2009; Chen et al. 2007). While there were differences in vaccine uptake between income categories in the survey population of adults aged 18–64 years without CMC, again, there was no clear relationship between increasing or decreasing income and vaccination status. No association between income level and non-vaccination was found in the high-risk adult groups. Given the smaller sample sizes in these target groups, it is possible that there was insufficient power to detect a statistically significant difference across income categories. More research is needed to better understand the underlying mechanisms for how these sociodemographic factors influence vaccination in order to develop effective strategies to increase uptake.

Although the survey was not sufficiently powered to allow for comparison of rates or determinants between all individual provinces and territories, we were able to assess potential associations between non-vaccination and jurisdiction based on the availability of public funding of the influenza vaccine. Lack of public funding was associated with lower influenza vaccine uptake in adults aged 18–64 years without CMC in QC as compared to other jurisdictions. However, no significant differences in non-vaccination were found between BC and NB as compared to the rest of Canada. Since the vaccine is not publicly funded for healthy adults aged 18–64 years in BC/NB  and 18-59 years in QC, this suggests that there are additional factors that explain the lower uptake of the influenza vaccine in this group other than public funding. In addition, in our analysis, cost was very rarely reported as a reason for non-vaccination among groups for which the influenza vaccine is not publicly covered (results not shown). A widely held misconception regarding vaccination is that a lack of public funding suggests that a vaccine is not necessary or important (Scheifele et al. 2014). Furthermore, due to time constraints or personal perceptions, health professionals may not regularly recommend vaccination to individuals for whom the vaccine is not publicly funded (Scheifele et al. 2014; Nichol and Zimmerman 2001). Additional research on how public funding of the influenza vaccine may influence perceived need can help inform promotional campaigns in addressing barriers to influenza vaccine uptake that are unrelated to vaccine cost.

Finally, the presence of CMC was associated with a lower risk of non-vaccination, both in participants aged 18–64 years and in those aged 65 years or more, and this association was independent from age. This finding is consistent with previous results from Canada (Polisena et al. 2012) and the US (Takayama et al. 2012). Those with chronic conditions generally have greater contact with the health system and providers, creating greater opportunity for vaccination and to receive a provider recommendation, which is an important determinant of influenza vaccine uptake (Polisena et al. 2012). The association between the presence of a CMC and higher influenza vaccine uptake may also be related to self-perceived health, although we did not assess this in the current survey. Individuals with CMC may perceive their health to be poorer and thus have a higher likelihood of receiving the vaccine due to a greater perceived, and actual, risk of complications from an influenza infection (Chen et al. 2007).

### Reasons for non-vaccination

A major framework that has informed research on health behaviours is the Health Belief Model (HBM) (Janz and Becker 1984; Coe et al. 2012; Nagata et al. 2013). In brief, the HBM proposes that the adoption of a particular health behaviour is influenced by a combination of factors, including (1) perceived risk of contracting a condition, (2) the perceived severity of the condition, (3) perceived benefits from a protective behaviour, and (4) the perceived negative aspects of this protective behaviour (Janz and Becker 1984).

In this survey, the most commonly stated reasons for not receiving the influenza vaccine were closely linked to the principles put forward by the model. The beliefs that individuals were not at high risk or that the influenza vaccine was not effective were the most commonly reported reasons in all adult groups, suggesting that perceived risk and the perceived benefits of vaccination may be low. Low perceived risk and lack of belief in vaccine effectiveness have been found to be consistently associated with lower uptake of the influenza vaccine (Nowak et al. 2015; Nagata et al. 2013).

Interestingly, a QC study of seasonal influenza vaccine uptake in 2011–2012 similarly found that low perceived susceptibility to, or severity of, the infection was the main reason for not receiving the vaccine, but doubts regarding vaccine efficacy were not frequently reported (Dubé et al. 2014).

### Limitations

There are a number of limitations to our analysis. First, the response rate for the survey was 19%. Response bias is possible as respondents may differ from those who declined to respond. No information for non-responders was available; therefore, we were unable to assess possible differences between those who were and were not captured in the survey and the potential for bias. Furthermore, we could not specify the number of respondents who were excluded for not knowing or refusing to report their vaccination status. Individuals who receive the influenza vaccine may be more likely to respond to surveys about immunization which may overestimate coverage (Centers for Disease Control and Prevention (CDC) 2013, 2016). However, our results were fairly consistent with findings from other Canadian studies that have utilized data from surveys with much higher response rates (Chen et al. 2007; Kwong et al. 2007). Recall bias is also possible since all data were self-reported. However, respondents were only asked to recall vaccination from less than 6 months prior, and several studies have demonstrated reliable recall for the seasonal influenza vaccine (Mac Donald et al. 1999). Furthermore, estimated rates of non-vaccination were similar to the results of other surveys in both Canada and the US. The analyses also relied on self-reported CMC status; therefore, there is the possibility of misclassification of respondents into target groups. However, given that the most common CMC of interest (e.g., asthma, chronic lung diseases, cardiovascular diseases) are well known, those affected are likely to be aware of their status and able to accurately report it. Nonetheless, misreporting of CMC was still possible. Finally, given the sample size of the survey, there was insufficient power to conduct more in-depth analysis of certain sociodemographic factors, particularly for adults aged 18–64 years with CMC (*n* = 408). Moreover, the sample size did not allow us to assess the KAB of respondents towards the influenza vaccine in relation to other sociodemographic factors, which would be of interest for informing promotional strategies and influenza vaccination campaigns.

## Conclusion

In general, the results of this analysis were consistent with the findings of other Canadian and American studies on the determinants of influenza vaccine uptake—particularly with regard to differences in non-vaccination in relation to age, sex, and chronic disease status (Takayama et al. 2012; Linn et al. 2010; Andrew et al. 2004; Chen et al. 2007; Kwong et al. 2007). Associations between income or education level and non-vaccination remain unclear; given the suboptimal influenza vaccine uptake in all risk groups in Canada, there is a clear need to better understand how these factors may influence influenza vaccine uptake in order to identify possible barriers to vaccination. The perception that the influenza vaccine is not needed was an important deterrent against the uptake of the influenza vaccine, even among high-risk adults in the survey. This information can inform efforts to raise awareness of potential complications from influenza infection and to address low confidence in the vaccine’s effectiveness.
